# Ossification of the Interosseous Membrane of the Leg in a Football Player: Case Report and Review of the Literature

**DOI:** 10.1155/2016/2930324

**Published:** 2016-01-06

**Authors:** Roberto Postacchini, Stefano Carbone, Marco Mastantuono, Carlo Della Rocca, Franco Postacchini

**Affiliations:** ^1^Section of Orthopaedic Surgery, Israelitic Hospital, Italian University of Sport and Movement, Rome, Italy; ^2^Department of Molecular Medicine, University “Sapienza”, 00100 Rome, Italy; ^3^Department of Radiology, University “Sapienza”, 00100 Rome, Italy; ^4^Department of Pathology, University “Sapienza”, 00100 Rome, Italy; ^5^Department of Orthopaedic Surgery, University “Sapienza”, 00100 Rome, Italy

## Abstract

*Introduction*. We report a case of ossification of the interosseous membrane (OIM) of the leg in a football player who had no history of severe local traumas. A review of the literature of the OIM of the leg in athletes was also carried out.* Case Report*. A 38-year-old Caucasian male patient complained of pain on lateral aspect of the leg when playing football. Pain progressively worsened until he had to stop the sporting activity. Radiographs, and then CT and MRI, showed OIM in the middle third of the left leg. MRI showed inflammation of tibia periosteum and bone adjacent to the ossification, which was then excised. Two months after surgery the patient returned to play football.* Conclusion*. A thorough analysis of the literature revealed three types of OIM of the leg in athletes. Type I usually occurs after a syndesmosis ankle sprain, Type II appears to result from a tibia fracture, and Type III, of which only one fully recorded case has been published, is probably caused, as in our patient, by repetitive minor traumas to the leg. Awareness of the existence of Type III OIM can avoid erroneous diagnoses leading to useless investigations and treatments.

## 1. Introduction

Heterotopic ossification consists in bone formation outside the skeleton. The most typical form of heterotopic ossification, unrelated to surgical procedures, neurologic injuries, or tumors, is myositis ossificans circumscripta, which usually occurs after a trauma but can even be initiated by multiple minor injuries or develop spontaneously [1]. Ossification of the interosseous membrane (OIM) of the leg may exhibit some features similar to those of myositis ossificans.

We report a case of OIM of the leg in a semiprofessional football player and analyse the different imaging, clinical, and pathogenetic features of the various types of this condition observed in athletes.

## 2. Case Report

A 38-year-old semiprofessional football player complained of pain in the lateral aspect of the left leg of 8-month duration. Initially, he had pain only for few minutes at the beginning of games. In the last 4 months, pain lasted for progressively longer periods of time until he had to stop playing. In the nonsporting activities there was no pain. After a period of rest, he returned to play but had to stop again due to persistent discomfort.

The patient had undergone excision of a left-sided L4-L5 disc herniation 4 years before, with no residual symptoms except for occasional episodes of back pain. When first seen, he reported having had an episode of back pain 1 month before. Physical examination showed no evidence of L5 radiculopathy. However, magnetic resonance imaging (MRI) of the lumbar spine was carried out on the suspicion of a recurrent L4-L5 disc herniation. MRI showed mild bulging at the operated on disc, which led to prescribing nonsteroidal anti-inflammatory medication for 3 weeks. Electromyography of the lower limbs was negative.

**Figure 1:**
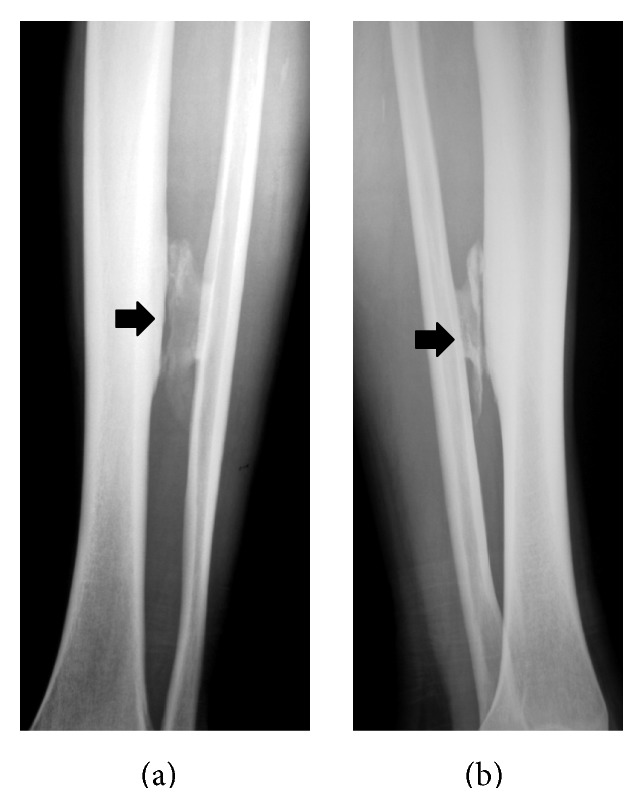
Anteroposterior (a) and lateral-oblique (b) radiographs of the left leg showing ossification in the middle third of the leg (*arrows*), possibly located in the interosseous membrane.

**Figure 2:**
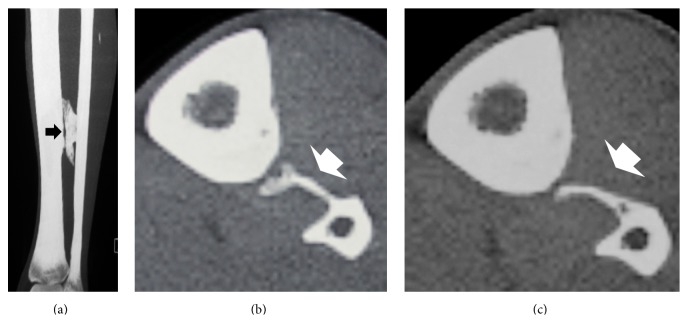
Coronal CT scan of the leg (a) demonstrates that the ossification (*arrow*) is in the interosseous membrane. It is in continuity with the fibula but separated from the tibia by a thin nonossified zone. Axial CT scans (b and c) show separation of the ossification (*arrows*) from lateral border of the tibia.

**Figure 3:**
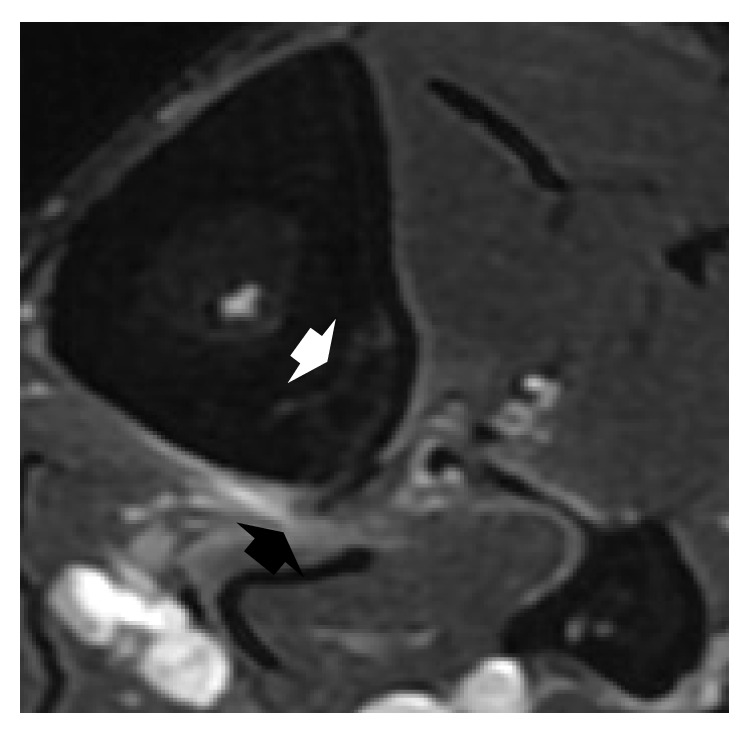
T2-weighted cross-sectional MR fat saturation sequence. The periosteum of the lateral margin of the tibia shows a hyperintense signal (*black arrow*) and hyperintense zones are visible in the lateral portion of the tibial diaphysis (*white arrow*).

When evaluated again, deep palpation on the middle third of the left peroneal muscles elicited moderate local pain. Both ipsilateral hip and knee appeared to be normal at clinical examination. Radiographs showed a heterotopic ossification in the middle third of the leg (Figure 1). The patient denied having sustained any severe trauma to the left leg. Computerized tomography (CT), performed to better determine the site and extension of the ossification, revealed that the lesion was located in the interosseous membrane and started from the left fibula but did not reach the lateral aspect of the tibia (Figure 2). MRI of the leg showed, on T2-weighted cross-sectional fat saturation sequences, a hyperintense signal on both the lateral aspect of the periosteum and lateral part of the tibial diaphysis at the level of the ossification (Figure 3). These findings indicated edematous changes, suggestive of a local inflammatory process that might be related to mechanical stress. The patient refused to undergo bone scan. Excision of the ossification was thus scheduled.

**Figure 4:**
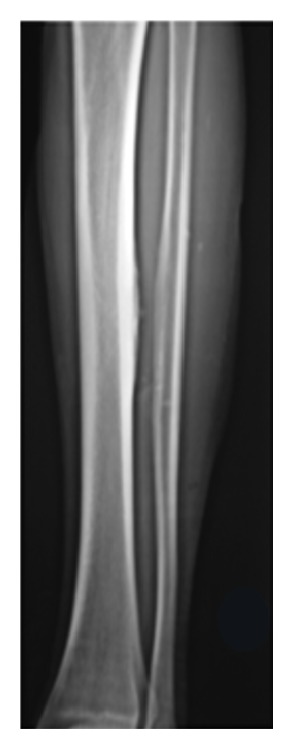
Postoperative anteroposterior radiograph showing complete excision of the ossification.

At operation, a posterolateral incision was carried out at the level of ossification. After longitudinal dissection and posterior retraction of the peroneus longus muscle, the fibular shaft was freed of muscle insertions. The tibialis anterior muscle was retracted anteriorly paying attention not to injure the interosseous artery and nerve. The lamina of heterotopic bone was cut from the fibula, elevated anteriorly to detach the insertions of the tibialis posterior muscle from its posterior aspect, and then excised. Postoperative radiographs confirmed complete removal of the lesion. Two months after surgery the patient returned to sporting activity with no discomfort. At 1-year follow-up, radiographs showed no recurrence of the ossification (Figure 4).

**Figure 5:**
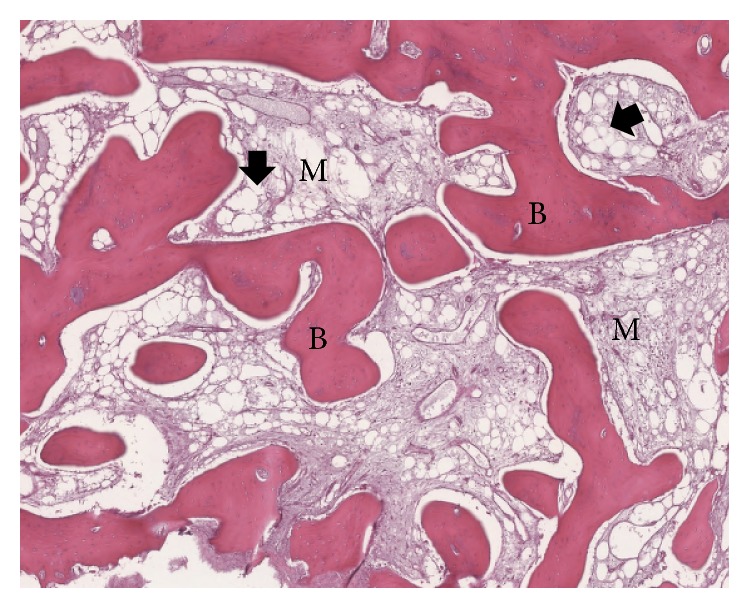
Low-power photomicrograph of the removed heterotopic ossification stained with hematoxylin-eosin showing mature lamellar bone (B) separated by medullary cavities (M) containing numerous areas of adipose tissue with expanded involution (arrows).

The excised specimen consisted of cortical bone arranged in slightly sclerotic trabeculae, focally in continuity with fibrous connective tissue. The bone marrow exhibited expanded adipose involution and mild fibrosis (Figure 5), as it may occur in some cases of myositis ossificans [1].

An informed consent was obtained from the patient for publication of the data.

## 3. Discussion

An extensive literature review allowed us to identify three different types of OIM of leg in subjects playing sport.

Type I, which is the most frequent, occurs at the distal end of the membrane as a result of a syndesmosis ankle sprain [2–5] or an inversion-internal rotation injury [6–8]. Overall, 28 cases were recorded, five of which showed a complete tibiofibular synostosis [4, 6–8]. The latter cases and one with incomplete synostosis [4] underwent surgical excision with no recurrence of the lesion except in one [7]. Type II was reported in three athletes who had a complete synostosis in the proximal or middle third of the membrane [9, 10]. In two, showing a large synostosis, the lesion was attributed to stress fracture of the tibia, whereas in the third, with a narrower ossification, the etiology was related to an old complete tibia fracture. Type III was first recorded in a short abstract as “interesting image” [11] providing no information on treatment and evolution of the lesion. The patient was a marathon runner with psoriasis who had been treated with a retinoid. The second patient [12] was a professional football player in whom the lesion was attributed to stress phenomenon to the interosseous membrane and resection of the ossification was performed, however, with no histological examination. Our case displayed clinical and imaging features completely similar to those of the two previous cases.

Type III OIM of leg has peculiar characteristics compared to the other two types. Ossification was located in the middle third of the membrane, started from the fibula and did not fuse completely with the tibia, was thin and few centimeters in height, and developed in the absence of severe traumas or stress fracture. Pain occurred during sporting activity and disappeared with rest and, in few months, progressively worsened. Furthermore, several investigations and/or treatments were consistently carried out before the correct diagnosis was made.

Pathogenesis of Type III OIM might be explained based on the theory of Chalmers et al. [13]. They postulated that in most heterotopic ossifications three factors are needed for the process to occur: an inciting event, usually represented by a trauma, a signal from the site of the injury, most likely represented by bone morphogenetic proteins secreted by the tissue cells, and an appropriate environment allowing the induction of newly formed bone. A possible explanation for the onset of ossification in our case, and possibly in the football player reported by James et al. [12], is that the inciting events were repetitive, minor, direct traumas on the lateral aspect of the leg, as often occurs in football players. In the marathon runner the inciting factor was presumably represented by the retinoid therapy, which is known to expose to heterotopic ossifications in connective tissue of several sites [14, 15]. The reason why all three patients had pain during sporting activity might be related to micromotion of the ossification, which did not fuse completely with the lateral aspect of the tibia, thus producing irritation of the periosteum and bone tissue of the lateral tibia. This interpretation is supported, in our case, by MRI findings suggesting an inflammatory process possibly dependent on mechanical stress to the periosteum and lateral tibia by the ossification.

In our case, surgical excision of the ossification resolved the patient's leg pain and allowed him to return to sport with no recurrence of the lesion, as what occurred for the patient of James et al. [12].

## 4. Conclusion

Three types of OIM of the leg have been described in athletes. Type I, the most frequent, occurs after a syndesmosis ankle sprain or an inversion-internal rotation injury. Type II, of which three cases were reported, was attributed to a stress or a complete fracture of the tibia. Only two cases of Type III have been reported so far. One of the two was the case of a football player, as our patient, who had to stop football due to pain in the involved leg. Excision of the ossification led to complete disappearance of pain.

## 5. Clinical Message

It is important to be aware of the existence of Type III OIM of leg in athletes to avoid the condition to be undiagnosed or misinterpreted with consequent inappropriate investigations and treatments.
